# Radiotherapy Versus Chemotherapy in Locally Advanced Cervical Cancer

**DOI:** 10.7759/cureus.44726

**Published:** 2023-09-05

**Authors:** Drashti Patel, Surekha Tayade, Vaishali P Tidke, Shikha Toshniwal, Hard Tilva

**Affiliations:** 1 Obstetrics and Gynecology, Jawaharlal Nehru Medical College, Datta Meghe Institute of Higher Education and Research, Wardha, IND

**Keywords:** external beam radiation, concomitant chemo-radiation therapy, locally advanced cervical cancer, radiotherapy (rt), cancer cervix

## Abstract

Eighty percent of women who have cervical cancer present at such an alarmingly advanced stage leading to high morbidity and mortality. Due to a lack of public awareness and inadequate infrastructure for screening and early identification in resource-poor countries like India, this tardy presentation is anticipated to continue in the future. Standard management for locally advanced squamous cell cervical cancer is radiotherapy. To increase responses and survival, neoadjuvant chemotherapy (NACT) was introduced to the arsenal. Recent studies from India have shown encouraging results for women getting concomitant chemo-radiation for locally advanced cervical cancer. However, toxicities are still a major problem. The approximated five-year actuarial survival rate with NACT is roughly 45% (95% confidence interval, 37-53%) with a median survival rate of 56 months. Compared to radiotherapy alone, patients receiving chemo-radiation are said to have a considerably better survival rate. Vomiting and nausea are the adverse effects that occur most frequently. Renal dysfunction and myelosuppression can also happen. However, there is evidence of effective tumor control. We will talk about a 55-year-old, para 5 elderly lady who had white discharge coming from her vagina and a cervical mass that bled when touched. She underwent NACT for six weekly cycles, followed by definitive chemo-radiation, and she responded favorably to this management strategy, indicating that the addition of chemotherapy is yet another cause for optimism in the management of cancer of the cervix.

## Introduction

Cervical cancer is the second succeeding cause of carcinoma-related deaths among women in India and the fourth-most prevalent cancer among women worldwide, with age-standardized incidence and mortality rates of 22 and 12.4 per 100,000 women per year, respectively [1]. India accounts for 25% of all cervical carcinoma-related mortality globally. Screening programs receive little support due to the distinct socio-cultural environment in Indian communities, lack of awareness, and insufficient infrastructure. Women frequently present to hospitals in the delayed stages of the illness despite having frequent vaginal discharge. For women with locally advanced cervical cancer (LACC), radiation therapy antiquated the preferred treatment method. However, numerous clinical trials and meta-analyses have shown that chemo-radiation can have a major impact on overall and progression-free survival while decreasing the risk of local and distant recurrences. The majority of these studies were conducted in India on women from industrialized countries, where disease and patient profiles are very different from place to place. Toxins, on the other hand, remain a major issue. A few trials from India have recently produced encouraging results in patients with LACC receiving concomitant chemoradiotherapy (CRT).

More research must be conducted and there must be experience in clinical settings, particularly in rural areas before concurrent chemo-radiation is incorporated into standard clinical practice. More studies and clinical experience are required, especially among rural patients, before concurrent chemo-radiation is integrated into standard clinical practice. Chemotherapy may be employed to treat both advanced cervical cancer and recurrent cervical cancer. Paclitaxel, topotecan, and cisplatin are indeed the three most commonly used chemotherapy drugs to treat cervical cancer. These drugs are also used in combination. Additional medications that can be used encompass docetaxel (Taxotere), ifosfamide (Ifex), 5-fluorouracil (5-FU), irinotecan (Camptosar), gemcitabine (Gemzar), and mitomycin. Bevacizumab (Avastin), an earmark medication, may be included in the list. These outcomes have not improved since the emergence of intensity-modulated irradiation 40 years ago, until this year [2]. We present a clinical case research to demonstrate how to manage locally advanced cancer therapy.

## Case presentation

In central India, a rural tertiary hospital received a report of a 55-year-old lady who had five children who had all been born at home vaginally. She had complained of vaginal white discharge for eight months, discomfort in the hypogastric area of the abdomen, and vaginal bleeding for one day. She had dropped 10 kg in the previous seven months and had intermittent breathlessness for the previous four months when she exerted herself. She appeared cachexia and was noticeably pallid upon general inspection. Clinical abdominal examination revealed no abnormalities, but local Sims' speculum inspection revealed an offensive vaginal discharge and a cauliflower-like growth measuring about 6 cm in size. Growth had taken over the entire cervix and had spread to the higher vagina as well. This growth was weak and oozed when touched. The clinical workup was supplemented with a bimanual inspection, and it was discovered that the parametrial tissue was affected all the way to the pelvic wall. The clinical finding was locationally advanced cervical cancer with a Stage 3 B FIGO (The International Federation of Obstetrics and Gynecology) staging.

**Figure 1:**
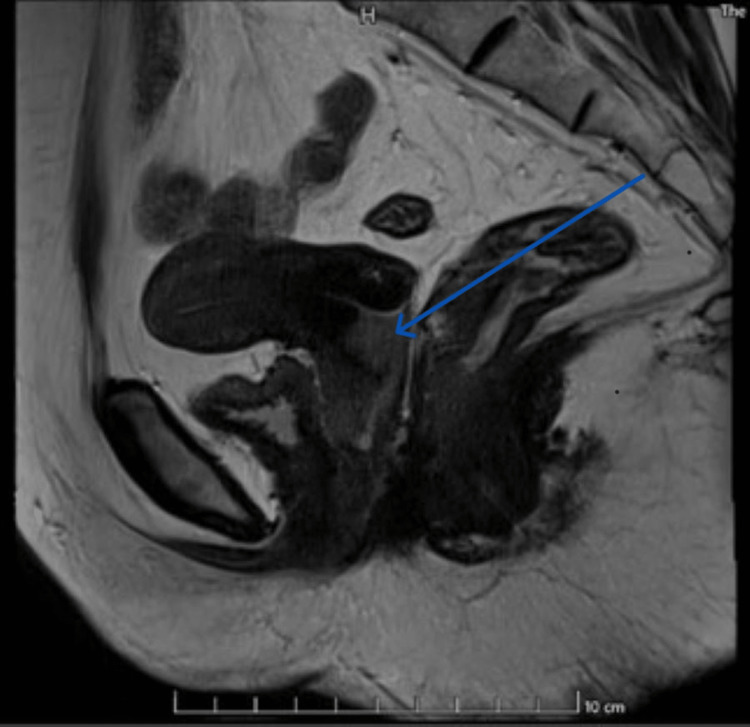
Magnetic Resonance Imaging-T2 Hyperintense Mass Lesion Cervical mass lesion showing no loss of fat plane between bladder and rectum.

On ultrasonography, the cervical area displayed an unclear hypoechoic structure with internal vascularity that was suggestive of a cancerous cervix. According to computed tomography, the cervix and the top third of the vagina were both affected by a heterogeneously enhancing soft tissue density lesion in a lower uterine segment. A neoplastic etiology was proposed after magnetic resonance imaging of the pelvis revealed a heterogeneously enhancing mass lesion that involved the lower uterine section, cervix, and upper third of the vagina (Figure 1). Punch biopsy results from the cervical mass were sent for histopathology, and slightly invasive squamous cell cervix cancer was diagnosed (well-differentiated type).

Following a cystoscopy, the posterior urinary bladder wall revealed irregularly elevated bladder mucosa with a trabeculated wall, raising the potential for tumor involvement in the mucosa. A team consisting of a gynecologist, one doctor, one surgeon, a pathologist, and a radiotherapist participated in the tumor board debate. It was determined that she should receive neoadjuvant chemotherapy (NACT) for 3-4 cycles weekly, followed by CRT because the disease was locally advanced and the mass was large.

The patient received day-care NACT consisting of six weekly doses of paclitaxel (60 mg intravenously over one hour) and carboplatin (AUC 5-6 intravenously over one hour), which was followed by chemo-radiotherapy in the form of external beam radiotherapy (EBRT), which was administered to the patient over a period of five and a half weeks. During EBRT, cisplatin (40 mg/m^2^) was given simultaneously once a week in accordance with recommended practices (EBRT). Within a week of the conclusion of EBRT, a microelectronic HDR device was administered brachytherapy (Elekta AB, Stockholm, Sweden). At weekly periods, a dose of 21 Gy was administered to the point in three subsequent fractions. Myelosuppression (anemia, thrombocytopenia, neutropenia), alopecia, fever episodes, and fatigue are adverse effects that the patient experienced. Gefitinib 250 mg once daily was administered to the patient as maintenance treatment. An examination of the pelvis and a contrast-enhanced CT scan of the abdomen and pelvis were used to measure the tumor response. During the first year, the patient was observed on an outpatient basis at three-month intervals, and then at six-month intervals.

## Discussion

Globally, a median of 37% of cervical cancer patients have localized advanced disease, though regional differences exist [3]. Studies from North America and Asia report lower and higher proportions of locally advanced disease, respectively, in comparison to other parts of the world. This may be because of variations in screening recommendations, access to healthcare, cultural factors influencing healthcare use, and limitations in the data sources and data collection methodologies.

Radiation therapy is the go-to therapy for women with LACC, which is categorized as stages IB3 to IVA as per the FIGO staging classification (version 2018) [4]. Internal radiotherapy and EBRT are routinely offered together. For about five weeks, daily X-ray treatments compensate for EBRT. Brachytherapy, either in a low-dose or high-dose regimen, is internal radiation. In a low dose regimen, 0.4-2 Gy of radiation/hour is given daily for 10 days, during which the patient stays in the hospital with intracavitary devices delivering the radiation to the diseased area. In a high-dose regime >12 Gy/hour dose is delivered at the point of maximum disease at weekly intervals for six weeks.

Innumerable research investigations throughout the years have suggested additional chemotherapy for better outcomes. To make the cancerous tumor cells more receptive to radiotherapy, this procedure employs low concentrations of chemotherapy drugs such as cisplatin (Platinol®). Locally advanced cervical carcinoma is usually treated with upfront radiotherapy coupled with a platinum-based regimen. The paracervical triangle's point A, that is, two centimeters above the external cervical os and two centimeters lateral to the uterus midline is defined by current RTOG (Radiotherapy Oncology Group) and GOG (Gynecologic Oncology Group) protocols as having received a cumulative dose of 80-90 Gy for cancer treated with definitive radiotherapy [1]. This correlates to the paracervical triangle, medial to the broad ligament, in which the uterine artery and uterine vein traverse the ureter. When compared to EBRT alone, the additional involvement of brachytherapy has exhibited superior rates of carcinoma-specific and cumulative survival benefits.

The biggest comparative study to date, conducted by Landoni, screened 343 patients with stage IB to stage IIA disorder (locally advanced), who underwent extensive surgery with pelvic lymph node dissection and were at risk for receiving radiation treatment if there was any residual disease [5]. The upfront radiation treatment was contrasted with this arm. No changes in survival were found, but there were significant variations in toxicity rates. The stage of the ailment had a substantial impact on the high survival advantage that chemo-radiation has been shown to provide. Additionally, the chance of recurrence is reduced by concomitant chemo-radiation.

The addition of cisplatin to radiotherapy was found to improve survival compared to radiation therapy alone in five studies. Following studies that eliminated hydroxyurea and compared upfront radiation therapy with concomitant cisplatin (with or without 5-Fluorouracil) with radiotherapy, which has been shown to increase survival and lower the risk of recurrence, and hence chemo-radiation became the standard management of medical care [6]. The addition of cisplatin to radiotherapy rationale is that it functions as a radiosensitizer, by inhibiting the Non-Homologous End Joining pathway, which is paramount in the Double Strand Breaks repair. Two Strands Radiotherapy uses high energy which causes DNA breaks. Every week, cisplatin is administered, and it must be administered carefully. Radiation therapy is provided a few hours before cisplatin. Before and after cisplatin infusion, sufficient hydration must be advised because it is a nephrotoxic agent. In addition to being aware of the patient's renal condition beforehand, dosing can be changed to avoid toxicity. It has been demonstrated that carboplatin can serve as an acceptable substitute for cisplatin in patients who cannot be administered with cisplatin infusion. However, cisplatin intolerance and abnormal renal function tests still make carboplatin the best option.

A large literature review of 18 trials consisting of chemo-radiation for LACC found that adding chemotherapy improved the average life span [6]. Chemotherapy is advised for women who have advanced or recrudesce diseases. Some drugs, such as cisplatin (Platinol) and paclitaxel (Taxol), are available. Five clinical trials found that concomitant CRT decreased the risk of death by 30-35% in LACC patients compared to radiotherapy (RT) alone. After completing synchronized CRT, the five-year overall survival (OS) rate for LACC patients is around 70%. Moreover, approximately 35% of patients continue to have cancer progression after CRT, and the outcome is still inadequate. In addition to CRT, NACT which is followed by surgical management is another alternative. Although both treatments improved cumulative survival, definitive CRT improved disease-free survival [6].

## Conclusions

The management of choice for LACC is chemoradiotherapy. The primary treatment for patients with LACC is still cisplatin combined with radiotherapy, whereas the treatment of metastatic and recurring cancer has changed more, with the addition of more advanced chemotherapeutic agents. The World Health Organization recently announced a global dynamism to eradicate cervical cancer within the next centennial. Till then, more work must be done to improve therapeutics and palliative care for women with cervical cancer.
